# Organ‐specific phytohormone synthesis in two *Geranium* species with antithetical responses to far‐red light enrichment

**DOI:** 10.1002/pld3.66

**Published:** 2018-08-21

**Authors:** Charlotte M. M. Gommers, Sara Buti, Danuše Tarkowská, Aleš Pěnčík, Jason P. Banda, Vincent Arricastres, Ronald Pierik

**Affiliations:** ^1^ Plant Ecophysiology Institute of Environmental Biology Utrecht University Utrecht The Netherlands; ^2^ Plant Development and Signal Transduction Program Center for Research in Agricultural Genomics (CRAG) Barcelona Spain; ^3^ Laboratory of Growth Regulators Centre of the Region Haná for Biotechnological and Agricultural Research Institute of Experimental Botany ASCR Faculty of Science Palacký University Olomouc Czechia; ^4^ Present address: Centre for Plant Integrative Biology School of Biosciences University of Nottingham Sutton Bonington UK

**Keywords:** auxin, brassinosteroids, *Geranium*, gibberellins, phytochrome signaling, shade avoidance

## Abstract

Plants growing in high densities experience a reduced red (R) to far‐red (FR) light ratio and shade‐intolerant species respond with accelerated elongation growth to reach the top of the canopy: the shade avoidance syndrome (SAS). FR‐enriched light inactivates phytochrome photoreceptors, which results in subsequent action of several plant hormones regulating growth. SAS is adaptive for shade‐intolerant plants, but is suppressed in shade‐tolerant plant species. Inspired by a previously published transcriptome analysis, we use two species of the genus *Geranium* here to study the involvement of auxin, brassinosteroids (BRs), and gibberellins (GAs) in supplemental FR‐induced elongation growth. *G. pyrenaicum*, a shade‐avoiding species, strongly induces auxin and gibberellin levels, but not BR, in elongating petioles. We show that, in this species, FR light perception, hormone synthesis, and growth are local and restricted to the petiole, and not the leaf lamina. Using chemical hormone inhibitors, we confirm the essential role of auxin and GAs in supplemental FR‐induced elongation growth. Shade‐tolerant *G. robertianum* does not display the change in hormone levels upon FR light enrichment, resulting in the lack of a shade avoidance response.

## 1 INTRODUCTION

Shade‐intolerant plants growing in dense stands, for example, a grassland or agricultural field, compete with neighbors to secure light interception. To do so, they enhance the elongation of stems and leaves, thus growing taller than surrounding plants. This adaptive trait is referred to as the shade avoidance syndrome (SAS) and largely depends on the modulation of the homeostasis of several plant hormones (reviewed in: Ballaré & Pierik, 2017; de Wit, Costa Galvão, & Fankhauser, 2016; de Wit, Keuskamp, et al., 2016). SAS is a favorable strategy for shade‐intolerant species, but it is suppressed by shade‐tolerant plants in the forest understory that are unable to outgrow neighboring trees (Gommers, Visser, St Onge, Voesenek, & Pierik, 2013; Gommers et al., 2017).

The reflection of far‐red (FR) and absorbance of red (R) light by green tissues causes a decline of the R to FR (R:FR) light ratio, which is perceived by the R and FR light‐sensitive phytochrome (Phy) photoreceptors of surrounding plants. Inactivation of Phy in low R:FR light releases the suppression of a group of basic helix–loop–helix (bHLH) transcription factors, the PHYTOCHROME INTERACTING FACTORS (PIFs). PIFs trigger the transcriptional cascade required for the elongation response, at least in part by regulating biosynthesis and signaling of several plant hormones (reviewed in Casal, 2013).

Auxins are often considered the major regulatory signals for phytochrome‐mediated shade avoidance. In *Arabidopsis thaliana*, exposure to low R:FR conditions induces auxin production in cotyledons or leaf laminas via transcriptional induction of *YUCCA* genes by PIF4, PIF5, and PIF7 (de Wit, Ljung, & Fankhauser, 2015; Hornitschek et al., 2012; Pantazopoulou et al., 2017). In addition, PIF4 and PIF5 induce the expression of several genes contributing to auxin responsiveness during low R:FR light (Hornitschek, Lorrain, Zoete, Michielin, & Fankhauser, 2009; Roig‐Villanova et al., 2007). Therefore, *Arabidopsis* mutants deficient in auxin biosynthesis, transport, or signaling are unable to induce low R:FR‐mediated hypocotyl and petiole elongation, leaf movement, and phototropism (Goyal et al., 2016; Keuskamp, Pollmann, Voesenek, Peeters, & Pierik, 2010; Michaud, Fiorucci, Xenarios, & Fankhauser, 2017; Nozue et al., 2015; Pantazopoulou et al., 2017; Tao et al., 2008).

Auxins coact with the steroidal plant hormones brassinosteroids (BRs) to induce elongation responses to FR enrichment (Kozuka et al., 2010) or blue light depletion (Keuskamp et al., 2011) in *Arabidopsis*. Although BRs biosynthesis is necessary for the response to end of day FR enrichment light (Kozuka et al., 2010), other studies report a reduction in BRs in shade‐grown *Arabidopsis* (Bou‐Torrent et al., 2014) or dark‐grown pea plants (Symons et al., 2002). BR sensitivity in low R:FR light conditions is enhanced via the transcription factors BR‐ENHANCED EXPRESSION (BEE) and BES1‐INTERACTING MYC‐LIKE (BIM) (Cifuentes‐Esquivel et al., 2013), and the PIF4–BRASSINAZOLE RESISTANT1 (BZR1) transcription factor complex (Oh, Zhu, & Wang, 2012), which induce elongation‐promoting pathways. In addition, BRASSINOSTEROID INSENSITIVE2 (BIN2), a BR signaling kinase, phosphorylates PIF4, which make BRs direct regulators of part of the light signaling cascade (Bernardo‐García et al., 2014).

Another group of PIF interactors are DELLA proteins, such as REPRESSOR OF GA1‐3 (RGA), GIBBERELLIN INSENSITIVE (GAI), and RGA‐LIKE1 (RGL1). DELLAs bind PIF4 and PIF5, inhibit their promoter binding ability, and thus suppress SAS (de Lucas et al., 2008; Feng et al., 2008). DELLA proteins are degraded under FR‐enriched light conditions due to enhanced action of bioactive gibberellins (GAs) (Djakovic‐Petrovic, de Wit, Voesenek, & Pierik, 2007). The GA biosynthetic genes *GA 20‐OXIDASE‐1* (*GA20ox1*), *GA20ox2*, and *GA3ox1* are transcriptionally induced by FR light enrichment, which leads to higher levels of GA_1_ and GA_4_, biologically active GAs (García‐Martinez & Gil, 2001; Reed, Foster, Morgan, & Chory, 1996).

As major regulators of plant development and growth, these phytohormones play a key role in regulation of SAS. Nevertheless, SAS is suppressed in plants that are unable to outgrow a shaded environment (Gommers et al., 2013). To date, it remains unknown how SAS is suppressed in such species and if auxins, BRs, and GAs play a role in this process. Our previous work has shown that two *Geranium* species from contrasting natural habitats (*G. pyrenaicum* and *G. robertianum*) display contrasting growth and gene expression patterns when exposed to FR‐enriched light conditions (Gommers et al., 2017). Here, we show that differential regulation of hormone levels correlate with enhanced elongation growth under FR enrichment in *G. pyrenaicum* and its absence in *G. robertianum*. In addition, we show that the site of FR light perception, hormone accumulation, and elongation is restricted to the petiole, and not to the lamina, of *G. pyrenaicum* leaves.

## 2 MATERIALS AND METHODS

### 2.1 Gene ontology enrichment analysis

For gene ontology analysis of previously published RNA sequencing data from Gommers et al. (2017) (Array Express E‐MTAB‐5371), significantly up‐ and downregulated *Geranium* OMCL groups upon FR light enrichment, with a BLAST E‐value <10^−10^ with *A. thaliana* genes, were clustered using the R package GOseq (Young, Wakefield, Smyth, & Oshlack, 2010), with correction for the total length of all transcripts in the *Geranium* OMCL group.

### 2.2 Plant material and growth conditions

For rosette experiments, *G. pyrenaicum* and *G. robertianum* seeds were sown and grown in long day conditions as described before (Gommers et al., 2017). Treatments started 2 weeks after transplanting.

For seedling experiments, seeds were surface sterilized in 70% EtOH followed by a 5% (*G. pyrenaicum*) or 10% (*G. robertianum*) bleach solution and rinsed with sterile water for four times. Seeds were sown on sterile agar plates (0.5 strength Murashige and Skoog medium, with 1 g/L MES buffer, 0.8% Plant Agar w/v) and stratified in the dark at 4°C for five (*G. pyrenaicum*) or seven (*G. robertianum*) days. Afterward, plates were transferred to long day light conditions, placed vertically in an angle of 70° to germinate for five (*G. pyrenaicum*) or eight (*G. robertianum*) days. Thereafter, seedlings were transferred to new agar plates, either or not with a pharmacological treatment. 24 hr later, light treatments would start.

### 2.3 Light treatments

FR‐enriched light conditions were obtained by supplementing standard growth chamber white light (WL, R:FR 1.8, 180 μmol m^−2^ s^−1^ PAR, Philips HPI 400 W) with far‐red LEDs (± 730 nm, Philips), to obtain a R:FR of 0.2 (WL + FR) without changing PAR (Gommers et al., 2017). Seedlings were exposed to a far‐red treatment for 5 (PEO‐IAA and NPA) or 4 days (PAC and BRZ), rosettes for 24 hr.

Local FR light treatments were applied using custom built light bundles, each containing four LED lights (diodes from Shinkoh Electronics) (Pantazopoulou et al., 2017). For FR_petiole_, the apical half of the petiole was enriched with FR light, and for FR_lamina_, the whole lamina was enriched with FR from below, to prevent illumination of other plant parts.

### 2.4 RNA isolation and gene expression

RNA extraction, cDNA synthesis, and RT‐qPCR were performed as described before (Gommers et al., 2017). The apical 1 cm of the petioles of the second leaf of three independent *Geranium* plants, were pooled as one biological replicate. RNA was extracted using the RNeasy kit (Qiagen) with on‐column DNAseI treatment, followed by cDNA synthesis using the Superscript III reverse transcriptase (Invitrogen) with RNAse inhibitors and random primers. Real‐time quantitative PCR (RT‐qPCR) was performed using Sybr Green Supermix (Bio‐Rad) in a Viia7 PCR. A list of the used primers is provided in Supporting Information Table S1. The *Geranium* orthologue for *AT3G57890* was used as a reference gene. Relative gene expression was calculated as 2^−ΔΔCT^.

### 2.5 Hormone analysis

For auxins, BRs, and GAs measurements, *Geranium* leaf lamina and petiole samples were harvested after 2 and 11.5 hr (12:00 and 21:30, respectively) of FR‐enriched (WL+FR) or control (WL) light treatment, as three biological replicates. These time points are identical to the ones used for transcriptomics in our previous study (Gommers et al., 2017).

For BRs content, these samples were analyzed as previously described (Tarkowská, Novák, Oklestkova, & Strnad, 2016) with a few modifications. In brief, fresh *Geranium* tissue samples of 50 mg were homogenized to a fine consistency using 3‐mm zirconium oxide beads (Retsch GmbH & Co. KG, Haan, Germany) and a MM 301 vibration mill at a frequency of 30 Hz for 3 min (Retsch GmbH & Co. KG, Haan, Germany). The samples were then extracted overnight with stirring at 4°C using a benchtop laboratory rotator Stuart SB3 (Bibby Scientific Ltd., Staffordshire, UK) after adding with 1 mL ice‐cold 60% acetonitrile and 10 pmol of [^2^H_3_]brassinolide, [^2^H_3_]castasterone, [^2^H_3_]24‐*epi*brassinolide, [^2^H_3_]24‐*epi*castasterone, [^2^H_3_]28‐norbrassinolide, [^2^H_3_]28‐norcastasterone, and [^2^H_3_]typhasterol as internal standards (OlChemIm Ltd., Olomouc, Czech Republic). The samples were further centrifuged, purified on polyamide SPE columns (Supelco, Bellefonte, PA, USA), and then analyzed by UHPLC‐MS/MS (Micromass, Manchester, UK). The data were analyzed using Masslynx 4.1 software (Waters, Milford, MA, USA), and BR content was finally quantified by the standard isotope dilution method (Rittenberg & Foster, 1940). Each sample was analyzed three times, and the average of these technical replicates was used as one biological replicate.

Auxins content in these samples was determined by slightly modified method described by Pěnčík et al. (2009). In brief, 10 mg of tissue was extracted with 1 ml cold phosphate buffer (50 mM; pH 7.0) containing 0.02% sodium diethyldithiocarbamate with internal standards: [^13^C_6_]IAA, [^13^C_6_]oxIAA, [^15^N,^2^H_5_]IAAsp, and [^15^N,^2^H_5_]IAGlu. Samples were then centrifuged at 36.000 *g* for 10 min, acidified with 1 M HCl to pH 2.7, and purified by solid phase extraction (SPE) using C8 columns (Bond Elut, 500 mg, 3 ml; Varian). After evaporation under reduced pressure, samples were diluted in 30 μl of 10% methanol and analyzed for auxin content using Acquity UHPLC™ (Waters, USA) linked to a triple quadrupole mass detector (Xevo TQ‐S™; Waters, USA). Each sample was analyzed three times, and the average of these technical replicates was used as one biological replicate. Deionized (Milli‐Q) water obtained from a Simplicity185 water system (Millipore, Bedford, MA, USA) was used to prepare all aqueous solutions. All other chemicals (analytical grade or higher purity) were from Sigma‐Aldrich Chemie (Steinheim, Germany).

GAs content in these samples was analyzed as described before by Urbanová, Tarkowská, Novák, Hedden, & Strnad, 2013; Shahnejat‐Bushehri, Tarkowska, Sakuraba, & Balazadeh, 2016.

### 2.6 Pharmacological treatments

For rosette experiments, different concentrations of 1‐naphthaleneacetic acid (1‐NAA (Duchefa Haarlem); 0.1, 1, 10, 50, 100, or 500 μM, in 0.1% EtOH, 0.1% Tween‐20), 24‐*epi*brassinolide (*epi*BL (Sigma‐Aldrich); 10, 50, or 100 μM, in 0.01% DMSO, 0.1% Tween‐20), and GA_3_ ((Sigma‐Aldrich) 10, 50, or 100 μM, in 0.01% EtOH, 0.1% Tween‐20) were sprayed on one leaf (petiole and lamina) of each plant (total 250 μL/leaf). Mock controls were either 0.01% DMSO, 0.01%, or 0.1% EtOH.

Different concentrations of auxin biosynthesis inhibitor yucasin ((Nishimura et al., 2013) 10, 25, or 75 μM, in 0.01% DMSO, 0.1% Tween‐20), auxin transport inhibitor 1‐*N*‐naphthylphthalamic acid (NPA (Duchefa Haarlem); 25 μM, in 0.1% EtOH, 0.1% Tween‐20), auxin perception inhibitor α‐(phenylethyl‐2‐one)‐IAA (PEO‐IAA (Hayashi et al., 2008); 50 μM, 0.01% DMSO, 0.1% Tween‐20), and BR biosynthesis inhibitor brassinazole (BRZ (Sigma‐Aldrich); 10, 50, or 100 μM, in 0.02% DMSO, 0.1% Tween‐20) were sprayed on the plants 24 hr prior to, and at the start of, the light treatment. For GA biosynthesis inhibition, pots were not watered for 2 days and then treated with 20 ml of paclobutrazol (PAC (Duchefa Haarlem); 100 or 500 μM, in 0.5% EtOH), or mock, 72 hr prior to the start of the light treatment. Mock treatments consisted of the appropriate concentration of solvent.

For seedling experiments, sterile solutions of PEO‐IAA (final concentration 10, 50, or 100 μM, 0.1% DMSO), NPA (final concentration 25, 50, or 100 μM, 0.1% DMSO), BRZ (final concentration 0.5 or 5 μM, 0.1% DMSO), or PAC (final concentration 10, 50, or 100 μM, 0.1% EtOH) were added to sterile agar medium. Mock plates contained the appropriate concentration of solvent. Seedlings were transferred to these plates 24 hr prior to the start of the light treatment.

### 2.7 Growth measurements

*Geranium* petiole elongation was measured over a given time with a digital caliper (t_x_–t_0_). Leaf laminas were harvested after 48 hours of light treatment and scanned (600dpi). The area was measured using ImageJ. In seedling experiments, cotyledon petioles were measured using the digital caliper at the end of the light treatment.

### 2.8 Statistical analysis

Differences between measurements were analyzed in Microsoft Excel with a Student's *t* test, preceded by an f test to check equal variances. Data in Figure 5 and Supporting Information Figure S5 are analyzed in R with a 1‐way ANOVA and a post hoc Tukey test, preceded by Levene's test, to check equal variances. Data was LN‐transformed if needed.

## 3 RESULTS

**Figure 1:**
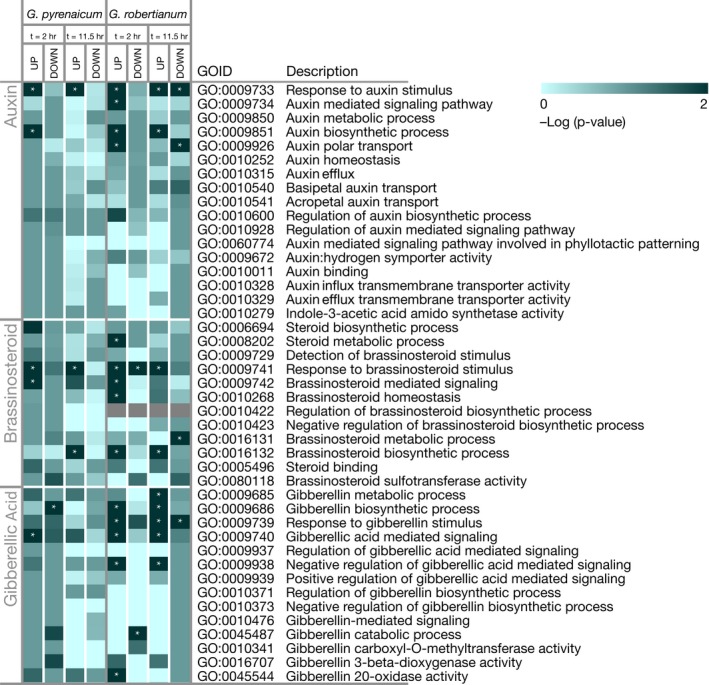
*Geranium pyrenaicum* and *G. robertianum* transcriptionally induce phytohormone‐related processes in FR‐enriched light. Heat map presenting (over‐) representation of Gene Ontology terms associated with auxin‐, brassinosteroid‐, and gibberellin‐related processes among up‐ and downregulated OMCL groups after 2 hr or 11.5 hr of FR‐enriched (R:FR 0.2) light in *G. pyrenaicum* and *G. robertianum*. Terms are clustered according to the different hormones. Data from the RNA sequencing study published in Gommers et al., 2017. Colors represent ‐Log of the *p*‐value, and significantly overrepresented terms (‐Log(p) > 2) are marked with asterisks

To get a first impression of auxin, BR, and GA regulation in response to FR enrichment in *G. pyrenaicum* and *G. robertianum*, we reanalyzed the RNA sequencing dataset previously published by Gommers et al. (2017). We applied gene ontology (GO) clustering to find (over) representation of biological processes among the up‐ and downregulated transcripts in *Geranium* petioles after 2 or 11.5 hr exposure to FR‐enriched light. A complete list of significantly enriched GO terms upon FR light enrichment in both species and both time points is presented in Gommers et al., 2017;. The heat map in Figure 1 shows the subset for auxin‐, BR‐, and GA‐associated gene ontology clusters. Several GO terms are significantly overrepresented among especially FR‐induced transcripts in both species.

**Figure 2:**
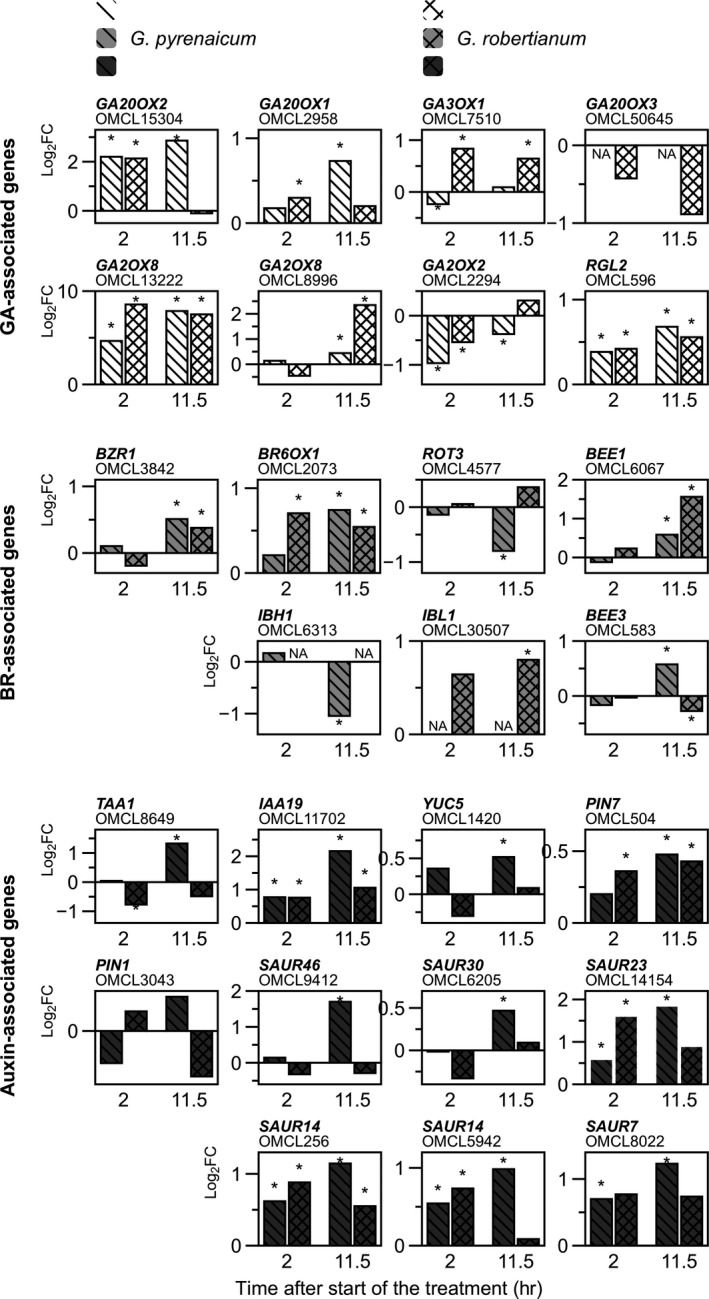
Expression patterns of gibberellin‐, brassinosteroid‐, and auxin‐associated OMCL groups in *Geranium pyrenaicum* and *G. robertianum* after 2 or 11.5 hr of FR‐enriched light. Log2‐transformed fold changes (WL + FR/WL) of OMCL families subtracted from the GO categories presented in Figure 1. Data from the RNA sequencing study published in Gommers et al., 2017. Asterisks mark significant induction/suppression (*p* < 0.01)

**Figure 3:**
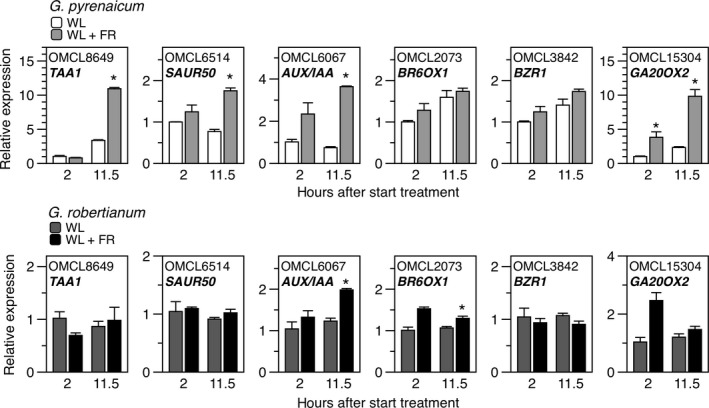
FR light enrichment affects hormone synthesis‐ and signaling‐associated gene expression in *Geranium pyrenaicum*, but less so in *G. robertianum*. Expression of *Geranium* orthologues of *TAA1*, an *AUX/IAA*,*SAUR50*,*BR6OX1*,*BZR1*, and *GA20OX2* in *G. pyrenaicum* (upper graphs) and *G. robertianum* (lower graphs) petioles upon 2 or 11.5 hr of FR light enrichment (WL + FR). Data are relative to the expression of a reference gene (orthologue of *AT3G57890*) and the WL control at 2 hr and represent means ± SE,* n* = 3 biological replicates. Asterisks indicate a significant difference between WL and WL + FR at the same time point, Student's *t* test, *p* < 0.05

Several FR light‐regulated genes are selected from these GO clusters and show clear differences in expression between the two species, as shown by more closely looking into the RNA‐Seq data (Figure 2) and by additional RT‐qPCR experiments (Figure 3). FR enrichment especially enhances transcription of GA synthesis gene orthologues (*GA20ox2*,* GA20ox1*,* GA3ox1*) in both species but to a lesser extent, specifically at the later time point, in *G. robertianum*, whereas this species did show stronger induction of GA catabolism gene orthologues (*GA2ox8*,* GA2ox2*) than *G. pyrenaicum*. Interestingly, the orthologue of BR synthesis gene *ROTUNDIFOLIA 3* (*ROT3*) is strongly suppressed in *G. pyrenaicum*, while *BR6OX1*, just like BR signal transducers *BZR1*,* BEE1*, and *BEE3* are marginally induced in the RNA sequencing dataset (Figure 2). This induction was not reproduced in the separate RT‐qPCR experiment in both species (Figure 3). Orthologues of negative regulators of BR signaling (*IBH1* and *IBH‐LIKE1* (*IBL*)) are suppressed in *G. pyrenaicum* but induced in *G. robertianum*. Transcripts for genes involved in auxin synthesis (*TAA1*,* YUC5*), transport (*PIN7*,* PIN1*), and signaling (*AUX/IAA* and several *SAUR* orthologues), are clearly more strongly induced by supplemental FR light in *G. pyrenaicum* than in *G. robertianum*, as shown by RNA‐Seq and RT‐qPCR data (Figures 2 and 3).

**Figure 4:**
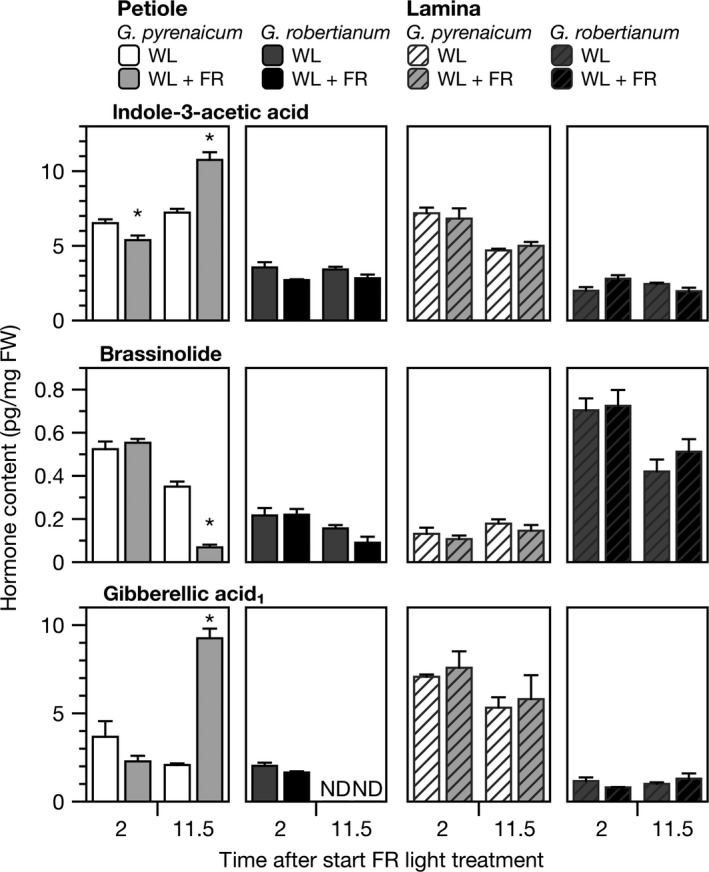
FR‐enriched light affects hormone levels in the *Geranium pyrenaicum* petiole, but not in the lamina, nor *G. robertianum*. Free indole‐3‐acetic acid (IAA), brassinolide (BL), and gibberellin A_1_ (GA_1_) levels (pg/mg fresh weight) in *G. pyrenaicum* and *G. robertianum* petioles (plain bars) and lamina (striped bars) exposed to 2 or 11.5 hr of control white light (WL; R:FR 1.8) or WL supplemented with far‐red light (WL + FR; R:FR 0.2). Data represent means ± *SEM*,* n* = 3 biological replicates. Asterisks represent significant differences in WL + FR compared to WL (Student's *t* test, *p* < 0.05). ND marks samples where no hormone was detected

This transcriptome analysis suggested that strong differences exist between the two species in regulation of hormone synthesis and signaling in response to FR enrichment. Next, we measured free auxin, BR, and GA levels in *G. pyrenaicum* and *G. robertianum* petioles and laminas after 2 or 11.5 hr of supplemental FR exposure. The data for the most active forms of all three hormone groups (indole‐3‐acetic acid, IAA; brassinolide, BL; and gibberellin A_1_, GA_1_) are presented in Figure 4. After 2 hr of FR light enrichment, no major differences were detectable in both species and tissues. After 11.5 hr, however, IAA and GA levels were strongly increased, while BL decreased, in *G. pyrenaicum* petioles. Interestingly, the hormone levels in *G. pyrenaicum* laminas were unaffected by the light treatment. In great contrast, in *G. robertianum*, IAA, BL, and GA_1_ levels were relatively low in petioles and generally unaffected by light treatment. More detailed analysis of several biosynthetic precursors and metabolites of all three hormone groups follow a similar pattern (Supporting Information Figures S1, S2, S3). Interestingly, multiple BL biosynthetic precursors showed great differences in absolute quantity between the species (Supporting Information Figure S2): C_27_ BRs originating from the cholesterol‐dependent biosynthesis pathway (28‐*nor*castasterone and 28‐*nor*teasterone, Joo et al., 2012) were more abundant in *G. pyrenaicum*, whereas C_28_ and C_29_‐BRs formed by the campesterol‐ and sitosterol‐dependent pathway (typhasterol and 28‐homocastasterone), respectively, were found to be more abundant in *G. robertianum*. As seen in Supporting Information Figure S3, several gibberellins were not detectable at all in both species (GA_4_, GA_7_), and some were only detectable in one of the treatments or time points. For example, the bioactive GA_5_ in *G. robertianum* was solely detectable in the tissues exposed to FR light enrichment.

**Figure 5:**
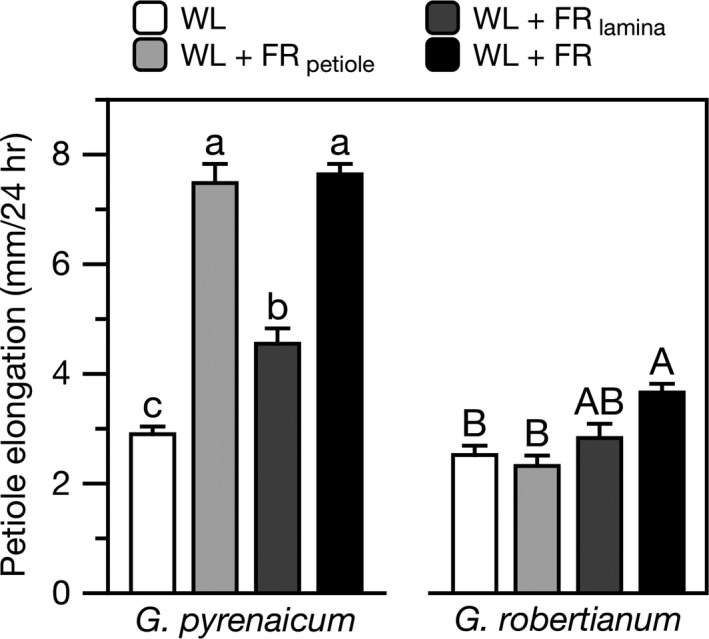
FR light illumination of the *Geranium* petiole, and not the lamina, induces elongation. Petiole elongation (mm 24 hr ^−1^) of *G. pyrenaicum* and *G. robertianum* plants grown in control white light (WL), WL supplemented with FR (WL + FR), or WL supplemented with a local FR treatment to only the petiole (WL + FR_petiole_) or the lamina (WL + FR_lamina_) for 24 hr. The treated leaf is measured. Data represent means ± *SEM*,* n* = 8 biological replicates. Different letters represent significant differences (1‐way ANOVA,* p* < 0.05)

The FR‐induced hormonal changes seem to be restricted to the petiole, which is also the organ that changes its growth in response to this light treatment, given that lamina size was not affected by FR enrichment (Supporting Information Figure S4) (Gommers et al., 2017). Next, we studied if this organ is also the required site of FR light exposure. Local application of FR light to the petiole‐induced elongation similar to FR applied to the whole plant in *G. pyrenaicum* (Figure 5). Interestingly, petiole elongation response was very weak when only the lamina, but not the petiole, of the same leaf was exposed to FR‐enriched light. The petiole elongation response was restricted to the FR‐exposed leaf and did not induce a systemic response in a younger leaf that did not receive FR enrichment (Supporting Information Figure S5). Petiole elongation in *G. robertianum* was completely unresponsive to FR enrichment of the petiole or lamina separately and showed a marginal response to whole‐plant FR enrichment, consistent with previous observation that this species hardly responds to FR enrichment (Gommers et al., 2017).

**Figure 6:**
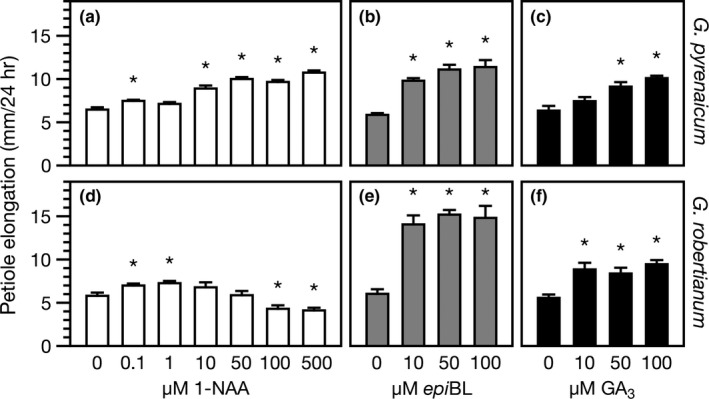
Auxin, brassinosteroid, and gibberellin induce growth in *Geranium pyrenaicum* and *G. robertianum* petioles. *G. pyrenaicum* (a–c) and *G. robertianum* (d–f) petiole elongation (mm 24 hr ^−1^) after treatment with different concentrations of 1‐naphthaleneacetic acid (1‐NAA; 0.1, 1, 10, 50, 100, 500 μM; a & d), 24‐*epi*brassinolide (*epi* BL; 10, 50, 100 μM; b,e), or gibberellic acid (GA_3_; 10, 50, 100 μM; c,f), which were sprayed on the petiole and lamina (total 250 μL/leaf). Data represent means ± *SEM*,* n* = 10 (1‐NAA) or *n* = 5 (*epi* BL and GA_3_) biological replicates. Asterisks represent significant differences with the mock control (0 μM) (Student's *t* test, *p* < 0.05)

We hypothesized that the lack of FR‐induced IAA, BR and/or GA synthesis could be responsible for the lack of petiole elongation in *G. robertianum* to FR enrichment. Therefore, we treated *G. robertianum* petioles with different concentrations of synthetic hormones under control (white‐) light conditions. *G. robertianum* responded with a clear petiole elongation response to all hormone treatments (Figure 6), but its auxin response at high concentrations was strongly reduced as compared to that of *G. pyrenaicum* (Figure 6a,d).

To verify whether the changes in endogenous hormone levels upon FR enrichment in *G. pyrenaicum* were essential for the petiole elongation response, we used chemical inhibitors to inhibit auxin synthesis (yucasin), perception (PEO‐IAA), transport (NPA), BR synthesis (brassinazole, BRZ), and GA synthesis (paclobutrazol, PAC). Application of these inhibitors in different concentrations to the *G. pyrenaicum* leaf (petiole and lamina; auxin inhibitors and BRZ) or potting soil (PAC) prior to and during the experiment had no effect on the petiole growth over 24 hr, both in control and FR‐enriched light (Supporting Information Figure S6).

To overcome the possibility that perhaps these chemicals would not be absorbed through the trichome‐rich epidermis of the *Geranium* leaf, we studied these chemicals in seedlings rather than mature plants. Unlike *Arabidopsis*, seedlings of both *G. pyrenaicum* and *G. robertianum* had nonresponsive hypocotyls in FR‐enriched light. Nevertheless, *G. pyrenaicum*, but not *G. robertianum*, showed a strong elongation response in the petioles of the cotyledons, and we used this response to assay shade and hormone responses in the *G. pyrenaicum* seedlings (Supporting Information Figure S7).

**Figure 7:**
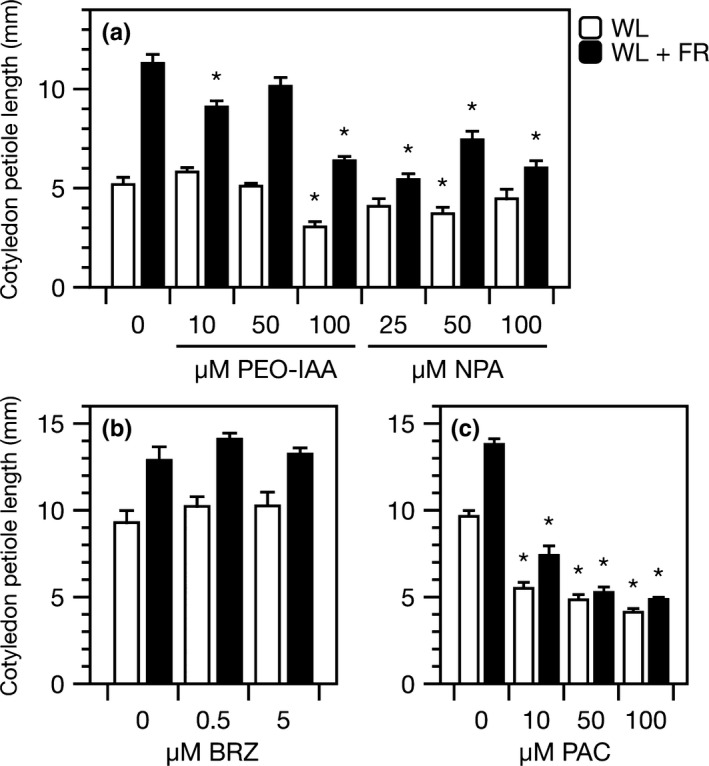
Inhibition of auxin or gibberellins, but not brassinosteroids, represses supplemental FR light‐induced elongation in *Geranium pyrenaicum*. Cotyledon petiole length of *G. pyrenaicum* seedlings after five (a) or four (b–c) days in control white light (WL; R:FR 1.8) or WL supplemented with far‐red light (WL + FR; R:FR 0.2), combined with different concentrations of hormone inhibitors: (a) α‐(phenylethyl‐2‐one)‐IAA (PEO‐IAA; inhibitor of auxin perception), 1‐*N‐*naphthylphthalamic acid (NPA; inhibitor of auxin transport), (b) brassinazole (BRZ; inhibitor of brassinosteroid synthesis), or (c) paclobutrazol (PAC; inhibitor of gibberellin synthesis). Chemicals were added to a medium, to which plants were transferred 24 hr prior to the start of the light treatments. Data represent means ±SEM,* n* = 6–10. Asterisks mark significant differences compared to mock in the same light treatment (Student's *t* test, *p* < 0.05)

Inhibition of auxin perception by PEO‐IAA and transport by NPA, inhibited the growth of *G. pyrenaicum* cotyledon petioles, especially in seedlings grown under FR‐enriched light (Figure 7a). This indicates that intact perception and distribution of this hormone is necessary for the SAS.

Even stronger growth inhibitions were visible in seedlings treated with PAC. Although elongation growth in general was inhibited, this effect was particularly strong for the supplemental FR light‐triggered growth responses, already by the lowest concentration. More severe treatments made the light effect disappear completely (Figure 7c).

Unlike IAA and GA, BR seems to play a less important role in the elongation responses to FR enrichment as BRZ hardly affected elongation growth in the seedling assays, irrespective of the light treatment (Figure 7b).

## 4 DISCUSSION

The plant hormones auxin, BR, and GA are well‐established regulators of shade avoidance responses (de Wit, Costa Galvão, et al., 2016). In the current study, we aimed to look for differences in hormone synthesis and signaling upon plant neighbor detection through FR light enrichment in two species with opposite growth strategies during shade. *G. pyrenaicum* is a shade‐intolerant species, often found in grasslands, and responds to simulated shade with strong petiole elongation. The closely related *G. robertianum* is a so‐called shade‐tolerant species that lacks the shade avoidance response. Reanalysis of a previously published RNA‐Seq dataset (Gommers et al., 2017), and additional RT‐qPCR analysis, confirms that supplemental FR light affects auxin‐, BR‐, and GA‐associated gene expression in both these species, similar to what was shown for *Arabidopsis* (de Wit, Spoel, et al., 2013; de Wit, Keuskamp, et al., 2016; Kohnen et al., 2016). The strong induction of auxin and GA_1_ synthesis genes in *G. pyrenaicum* are consistent with increased levels of these hormones in FR‐enriched light, while in *G. robertianum* both the gene expression patterns and auxin and GA_1_ levels are hardly affected by the light treatment. Remarkably, another biologically active gibberellin, GA_5_, was undetectable in control white light, but abundant in FR light‐treated petioles of *G. robertianum*. In this species, this induction clearly does not lead to enhanced petiole elongation. Consistent with this observation, an enrichment of GA_1_ in the shade did also not positively correlate with hypocotyl growth in a non‐shade‐avoiding *Arabidopsis* mutant *shadow‐1* (Li et al., 2016).

Although hormone‐related gene expression patterns already change soon (2 hr) after the start of the shade treatment, hormone abundance changes later. In *Arabidopsis* seedlings, auxin and CS levels rapidly change after FR light exposure, but GA levels increase only after prolonged treatments (Bou‐Torrent et al., 2014; Tao et al., 2008). In addition, genes involved in hormone‐signaling pathways are rapidly upregulated upon exposure to FR‐enriched light (Kohnen et al., 2016). Our data might indicate that in *Geranium*, early FR light‐induced petiole growth would be associated with enhanced sensitivity to the already present pool of hormones (via enhanced expression of signaling genes) and that changing hormone levels would promote petiole growth in prolonged FR treatments.

Interestingly, unlike *Arabidopsis*, FR‐induced IAA accumulation in *G. pyrenaicum* is restricted to the petiole, and not the lamina (de Wit et al., 2015). Consistent with our differential auxin accumulation data showing accumulation in the petiole, also FR detection in the petiole specifically is required for the elongation response in *G. pyrenaicum* (Figure 5). In *Arabidopsis*, the elongation response is induced solely by FR light perceived in the petiole as well (Pantazopoulou et al., 2017), while lamina‐perceived FR light induces local auxin synthesis in the lamina, probably followed by transport toward the petiole and ultimately leading to upward petiole movement (hyponasty) (Michaud et al., 2017; Pantazopoulou et al., 2017). *G. pyrenaicum* does not induce hyponasty in response to FR enrichment (data not shown), possibly because of the putative absence of FR‐triggered auxin synthesis in the lamina in this species.

The important role for auxin in supplemental FR‐induced petiole elongation is supported by (a) the lack of IAA synthesis and IAA signaling gene expression as well as growth response in FR‐treated *G. robertianum* and (b) the inhibition of a petiole elongation response to FR enrichment in *G. pyrenaicum* seedlings treated with auxin transport‐ and perception inhibitors.

Even though auxin is considered to be the key player mediating supplemental FR‐induced shade avoidance (de Wit, Lorrain, & Fankhauser, 2013), our data show that GA levels also strongly increase in *G. pyrenaicum* petioles when exposed to FR enrichment. Similar to auxin, GA levels also increase specifically at the site of perception and response, the petiole (Figure 5, Supporting Information Figure S4). Albeit to a lesser extent, GA_4_ levels were found to be induced in whole *Arabidopsis* seedlings exposed to far‐red light (Bou‐Torrent et al., 2014), consistent with increased expression of *GA20ox* genes (Hisamatsu, King, Helliwell, & Koshioka, 2005). Upon binding of GA to its receptor GID1, DELLA proteins are degraded in low R:FR light conditions, and this releases the DELLA‐mediated inhibition of PIFs and thus shade avoidance (de Lucas et al., 2008; Djakovic‐Petrovic et al., 2007; Murase, Hirano, Sun, & Hakoshima, 2008). Consistent with this growth‐promoting role for GA, petiole elongation of both *Geranium* species is enhanced by exogenous GA_3_ application (Figure 6) and the supplemental FR‐induced elongation of cotyledon petioles in *G. pyrenaicum* is prevented upon chemical inhibition of endogenous GA synthesis (Figure 7). Importantly, the lack of additional GA_1_ accumulation in *G. robertianum* under FR‐enriched light corresponds with its lack of a petiole elongation response to this light treatment.

The observation that *G. robertianum* and *G. pyrenaicum* can both respond to exogenous IAA and GA treatments and indicates that the differences in shade avoidance between these are more likely occurring at the level of local hormone synthesis than at the level of hormone response.

Unlike IAA and GA, bioactive BRs brassinolide (BL) and castasterone (CS) were reduced upon FR light enrichment in *G. pyrenaicum* petioles. This result seems contradictory, as BRs are growth‐promoting substances (Figure 6) and earlier studies have shown that functional BR synthesis is essential for the full growth responses to shade cues (Keuskamp et al., 2011; Kozuka et al., 2010). Interestingly, other studies to measure CS levels in low R:FR‐exposed plants found a similar reduction in *Arabidopsis* (Bou‐Torrent et al., 2014) and unchanged levels in sunflower seedlings (Kurepin, Joo, Kim, Pharis, & Back, 2012). Furthermore, dark‐grown pea seedlings that elongate rapidly had reduced CS levels compared to light‐grown controls (Symons et al., 2002). Perhaps, petiole elongation responses depend more strongly on BR signaling than on their biosynthesis. Light‐ and brassinosteroid signaling are known to converge at the level of the PIF4 transcription factor complex. Low R:FR light‐stabilized PIF4 interacts with the BR‐activated transcription factor BZR1 and, among others, enhances auxin signaling, GA biosynthesis, and DELLA suppression (Oh et al., 2012, 2014; Shahnejat‐Bushehri et al., 2016).

**Figure 8:**
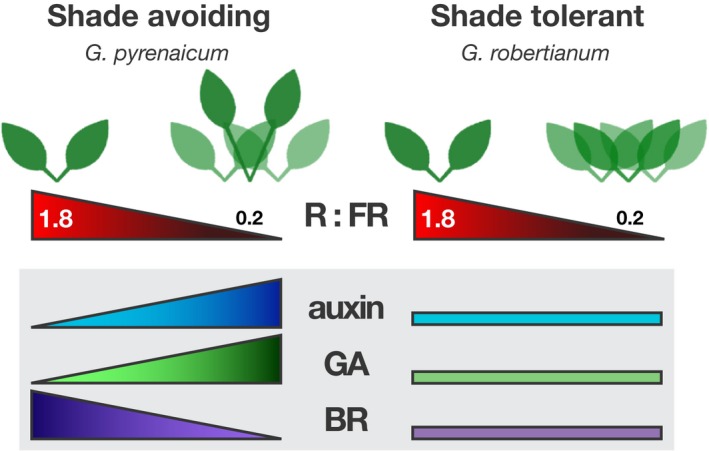
Graphic summary. When plants grow in dense communities, the R:FR light ratio decreases through R absorption and FR reflection, which is perceived in the petioles and causes a local increase in auxin and gibberellin (GA), but a reduction in brassinosteroid (BR) in shade‐avoiding plant species *Geranium pyrenaicum* (left). These changing hormone levels regulate the elongation of petioles, which facilitates shade escape. When shade‐tolerant species *G. robertianum* (right) is exposed to FR‐enriched light, this does not affect hormone synthesis or signaling, which results in no elongation response in its petioles

To conclude, our analysis of transcription patterns, hormone synthesis, and growth in FR‐enriched light environments show that local light perception and synthesis of auxin and GA, but not BR, account for the SAS in the shade‐intolerant *G. pyrenaicum*. Likewise, the lack of a FR light effect on hormone levels in shade‐tolerant *G. robertianum* seems responsible for its lack of the SAS (Figure 8). Future studies could be focused at identifying if the previously identified differential regulation of the atypical bHLH protein KIDARI upon FR light enrichment (Gommers et al., 2017) is functionally associated with the differential hormone patterns described here between these two species.

## AUTHOR CONTRIBUTIONS

CG and RP conceived and designed the project. CG, SB, DT, AP, JB, and VA performed the experiments and analyzed the data. CG and RP wrote the manuscript. All authors discussed the data and gave substantial comments on the manuscript.
