# Supplementary material for: Organ‐specific phytohormone synthesis in two Geranium species with antithetical responses to far‐red light enrichment
Source: Plant Direct. 2018 Aug 21;2(8):e00066. doi: 10.1002/pld3.66 (PMC6508794; doi:10.1002/pld3.66)
Supplement: Supplementary file 1 [file PLD3-2-e00066-s001.pdf]

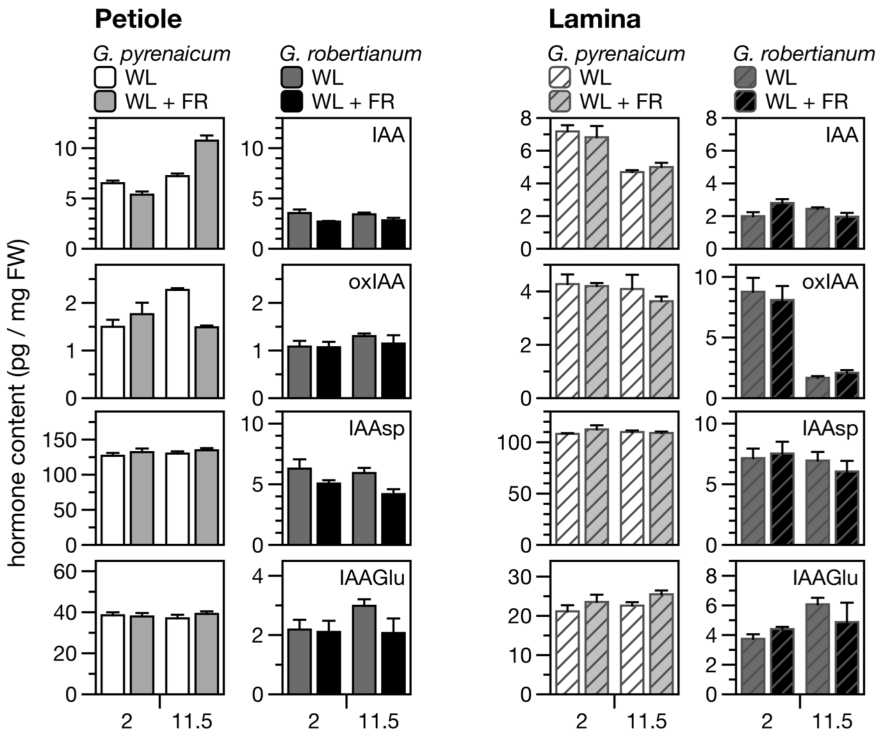

Figure S1. Levels (pg / mg fresh weight) of free IAA, catabolite oxIAA and conjugates IAAsp and IAAGlu in *G. pyrenaicum* and *G. robertianum* petioles (plain bars) and lamina (striped bars) exposed to 2 or 11.5 hours of control white light (WL; R:FR 1.8) or WL supplemented with far-red light (WL + FR; R:FR 0.2). Data represent means ± SEM, n = 3 biological replicates.

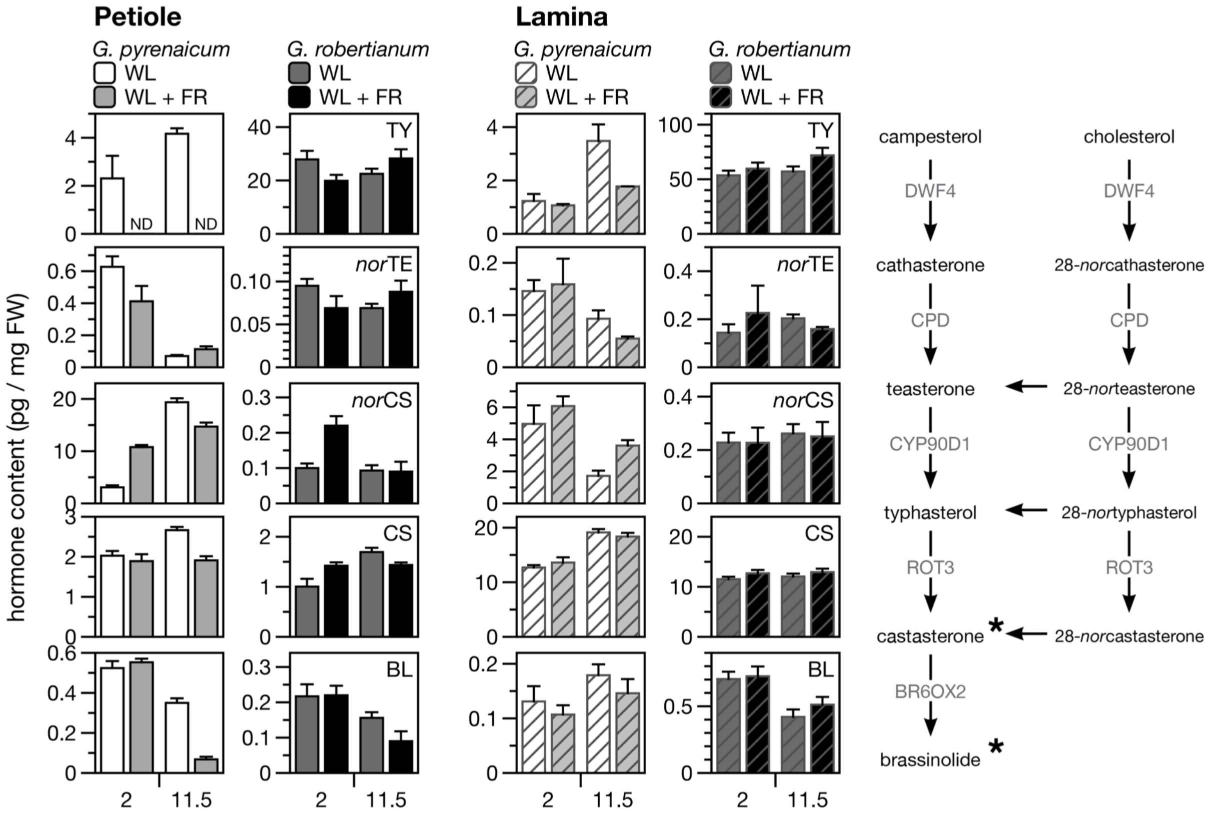

Figure S2. Levels (pg / mg fresh weight) of several brassinosteroids (typhasterol (TY), 28-norteasterone (*nor*TE), 28-norcastasterone (*nor*CS), castasterone (CS) and brassinolide (BL)) in *G. pyrenaicum* and *G. robertianum* petioles (plain bars) and lamina (striped bars) exposed to 2 or 11.5 hours of control white light (WL; R:FR 1.8) or WL supplemented with far-red light (WL+FR; R:FR 0.2). Data represent means ± SEM, n = 3 biological replicates. On the right side, a simplified pathway of BR biosynthesis, with bioactive BRs marked by asterisks the associated enzymes marked in grey.

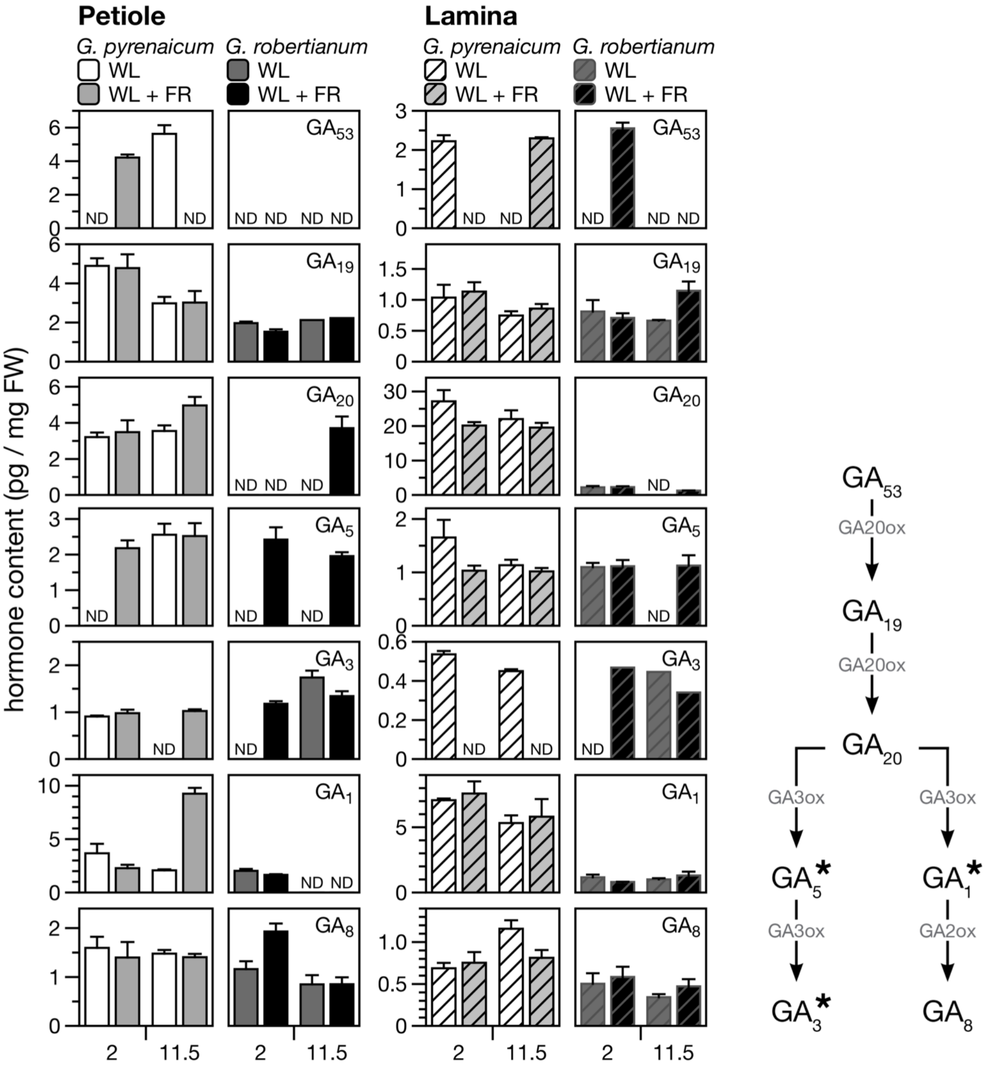

Figure S3. Levels (pg / mg fresh weight) of several gibberellins (GA_{53}, GA_{19}, GA_{20}, GA_5, GA_3, GA_1 and GA_8) in *G. pyrenaicum* and *G. robertianum* petioles (plain bars) and lamina (striped bars) exposed to 2 or 11.5 hours of control white light (WL; R:FR 1.8) or WL supplemented with far-red light (WL + FR; R:FR 0.2). Data represent means ± SEM, n = 3 biological replicates. On the right side, a simplified pathway of GA biosynthesis, with bioactive GA_1, GA_5 and GA_3 marked by asterisks and the associated enzymes marked in grey.

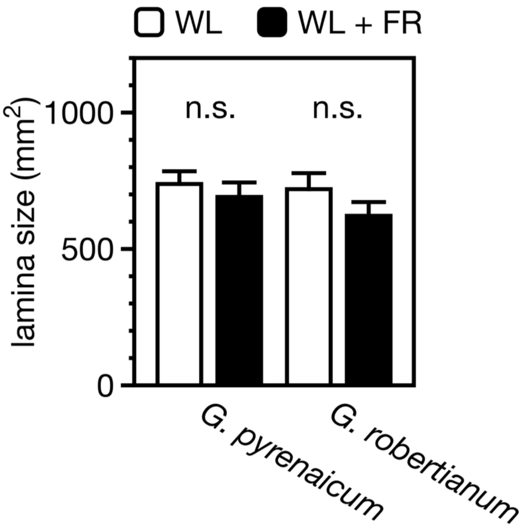

Figure S4. FR light enrichment does not affect *Geranium* lamina size.
Lamina size (in mm^2) of G. pyrenaicum and G. robertianum leaves grown in control white light (WL; R:FR 1.8) or WL supplemented with far-red light (WL + FR; R:FR 0.2) for 48 hours. Data represent means ± SEM, n = 7 biological replicates. No statistical differences are found (student's t-test, $p < 0.05$).

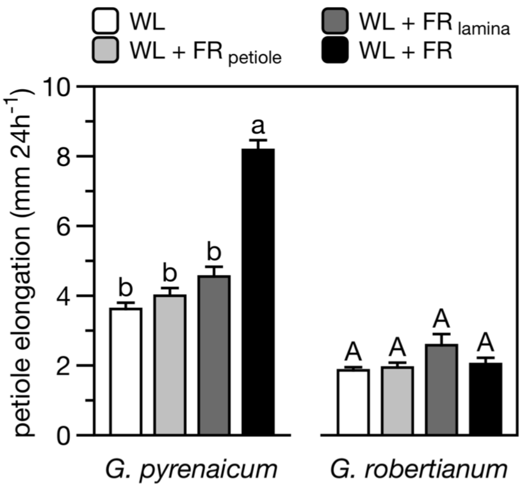

Figure S5. FR light illumination of *Geranium* petioles does not induce a 'systemic' response in non-treated leaves.

Petiole elongation (mm 24 h^{-1}) of *G. pyrenaicum* and *G. robertianum* plants grown in control white light (WL), WL supplemented with FR (WL + FR), or WL supplemented with a local FR treatment to the petiole (WL + $FR_{petiole}$) or the lamina (WL + FR_{lamina}) of an older leaf of the same plant, for 24 hours. Measurements of a younger ('systemic') leaf during the same experiment as presented in Figure 2B. Data represent means ± SEM, n = 8 biological replicates. Different letters represent significant differences (1-way ANOVA, $p < 0.05$).

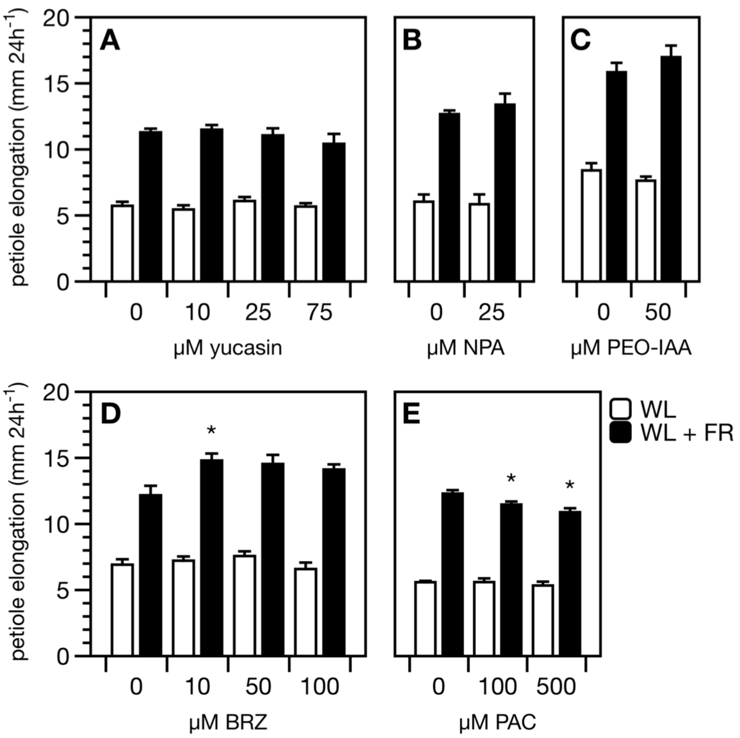

Figure S6. Inhibition of auxin, brassinosteroids or gibberellins has no effect on supplemental FR light–induced elongation in *G. pyrenaicum* petioles.

Petiole elongation (mm 24 h^{-1}) of *G. pyrenaicum* plants grown in control white light (WL; R:FR 1.8) or WL supplemented with far-red light (WL + FR; R:FR 0.2), combined with different concentrations of hormone inhibitors: (**A**) yucasin (inhibitor of auxin synthesis), (**B**) 1-*N*-naphthylphthalamic acid (NPA; inhibitor of auxin transport), (**C**) α-(phenylethyl-2-one)-IAA (PEO-IAA; inhibitor of auxin perception), (**D**) brassinazole (BRZ; inhibitor of BR synthesis) or (E) paclobutrazol (PAC; inhibitor of GA synthesis). Yucasin, NPA, PEO-IAA and BRZ were sprayed (250 µL) on the plants 24 h before, and at the start of the light treatment (10:00 AM). PAC was applied to the soil, 72 h before the start of the light treatment. Data represent means ±SEM, n = 6-7. Asterisks mark significant differences compared to mock in the same light treatment (student's t-test, $p < 0.05$).

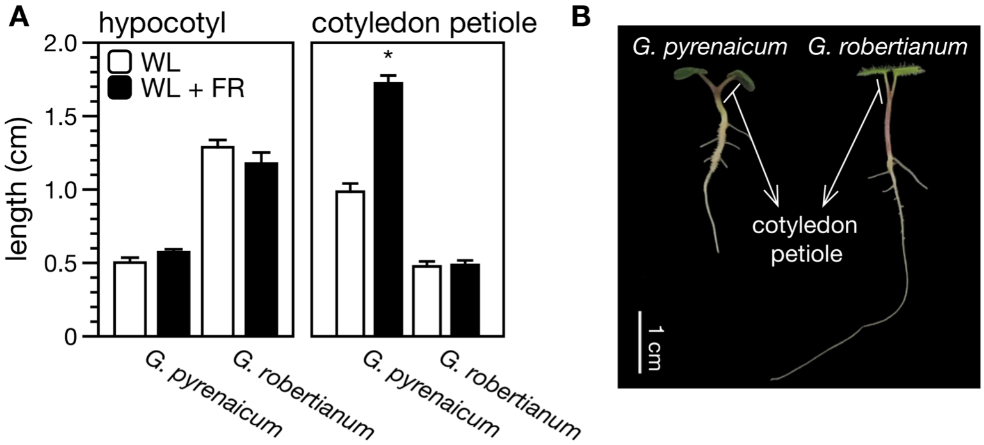

Figure S7. The shade avoidance syndrome in *Geranium* seedlings is restricted to cotyledons. (**A**) Hypocotyl- and 'cotyledon petiole' length of 5 day old *G. pyrenaicum* and *G. robertianum* seedlings grown in control white light (WL; R:FR 1.8) or WL supplemented with far-red light (WL + FR; R:FR 0.2). Data are means ± SEM, n = 6 – 8 biological replicates, asterisk indicates significant difference between WL and WL + FR (student's T-test, $p < 0.05$). (**B**) picture of *G. pyrenaicum* (left) and *G. robertianum* seedling, indicating which organ is referred to as the 'cotyledon petiole'.

Table S1. Primers used in this study

species	OMCL	orthologue	LP	RP
G. pyrenaicum	OMCL1725	*AT3G57890 (reference)*	TGCAGGGTGAGTTAACGAGT	TGAAGTTGGCATTGACGCAA
G. robertianum	OMCL1725	*AT3G57890 (reference)*	ATCCGAGTGCAAAATCGACG	ACTCGATTCTGCAGCCATCT
G. pyrenaicum	OMCL8649	*TAA1*	GACTCCTCACACTCCAACCA	TCAGCCTCCCTCAGTACTCT
G. robertianum	OMCL8649	*TAA1*	GCGTGTATTGTGGCCAGTAG	TGACATCCCCGAACAATCCA
G. pyrenaicum	OMCL6514	*SAUR50*	AAGCTATCACAAGGGGCTGT	CTCGTACCCACCGTTCTTCT
G. robertianum	OMCL6514	*SAUR50*	TGTGGATGGGTCATGGTCAA	AGGAGGAAGCAAGAGAAGCA
G. pyrenaicum	OMCL6067	*AUX/IAA*	TTTCACGTACTTAGTCGCCG	ATCAGTACCTCGACGTCTGT
G. robertianum	OMCL6067	*AUX/IAA*	CATTTTCCATGGCACGTCAC	AATGGACCAAGACAACGGAG
G. pyrenaicum	OMCL2073	*BR6OX1*	CTCGGGTCGTTTTCCTTAGA	TGGCAATCAGGGAGAGAAAA
G. robertianum	OMCL2073	*BR6OX1*	GAAGCGAGAGAGTACCCAAA	ATTGGGATGGAAAGGTCGTC
G. pyrenaicum	OMCL3842	*BZR1*	AGAGCTGCAGAGTTTGAGTT	TCTTAGCTCCGAGTTTTCCC
G. robertianum	OMCL3842	*BZR1*	CTAGCACTCTCTGTCATGGC	GTTCCCACCTCTCCTACTTA
G. pyrenaicum	OMCL15304	*GA20OX2*	ATTACCACGCACACTCCCTT	CGGGCCTGATTTTGAACCAA
G. robertianum	OMCL15304	*GA20OX2*	CTGCCACTTGTTTTCGGTGA	TCAGGATTGACACTCGGGAC
