# Supplementary material for: Organ‐specific phytohormone synthesis in two Geranium species with antithetical responses to far‐red light enrichment
Source: Plant Direct. 2018 Aug 21;2(8):e00066. doi: 10.1002/pld3.66 (PMC6508794; doi:10.1002/pld3.66)
Supplement: Supplementary file 2 [file PLD3-2-e00066-s002.pdf]

Reviewer#1:

Gommers et al performed reanalysis of previously published transcriptome data and observed over representation of various hormone biosynthetic/signaling genes under shade conditions. They focused on auxin, GA and BR pathways and found that while auxin and GA biosynthetic pathways are upregulated in response to shade in a shade sensitive species, BR biosynthesis was downregulated in this species. Strikingly, the hormone biosynthesis and signaling pathways were unaffected in a shade tolerant species under shade conditions. They measured hormone levels, identified the site of shade perception and performed pharmacological intervention to demonstrate the difference between the shade sensitive and tolerant species. Overall, this is an excellent study and enhances our understanding of SAS response. This study is well-designed and well written. Just one suggestion to enhance of understanding.

While the hormone levels change in a shade sensitive species while it doesn't change in shade tolerant species, the authors didn't ask why it didn't change. We now know most of the regulatory genes necessary to make this change. Perhaps, the authors should focus on those in a comparative study in both shade sensitive and tolerant species to dissect why the hormone levels didn't change in the shade tolerant species. This would add strength to the manuscript and clarify better difference between the two species.

To stress this comparison between the (lack) of enhanced gene expression and hormone levels more clearly, we have performed additional RT-qPCR analysis on hormone-associated genes (new Fig. 3) and added a sentence (starting at line 353) that bridges the gene expression data from the RNAseq analysis and the (newly added) RT-qPCR data and the hormone measurements. We see that *Geranium* orthologues of genes involved in auxin synthesis (*TAA1*) and GA synthesis (*GA20OX2*) are up-regulated in WL+FR in *G. pyrenaicum*, but less so in *G. robertianum*. This correlates with the levels of IAA and GA presented in figure 3. The expression of BR biosynthesis gene *BR6OX1*, as well as *BZR1* were upregulated by FR enrichment in the RNA seq data, but this could not be confirmed by RT qPCR. This result also correlates with the lack of BR increase upon FR light exposure. We have added these new results to the manuscript accordingly.

Sentence 372-373: indicate that the hormone signaling is not limiting in these species, but the difference in shade avoidance might be due to a difference in hormone biosynthesis. But, the auxin signaling in 5A vs 5D shows a strong difference between the two species. I wonder if the authors have looked at the expression of any auxin signaling genes in these species.

The data in (now) Fig. 6 (formerly Fig. 5) show a difference in auxin responsiveness between these two species. However, this experiment is performed in WL without supplemental FR light. The gene-expression data indicate that *G. robertianum* does express genes involved in auxin signaling, similar to *G. pyrenaicum* (and *Arabidopsis*), but these genes are not strongly induced by supplemental FR light (Fig. 2 and Fig. 3). Based on our data we

suggest that the differences in figure 6 are not caused by a lack of auxin signaling components in *G. robertianum*. Nevertheless, the lack in shade-induced growth in this species might be caused by the lack of FR-enhanced expression of these genes. We have added this lack of FR-sensitivity of auxin signaling components in *G. robertianum* as data that supports the major role of auxin in FR-induced growth (sentence 394).

Reviewer#2:

Gommers et al. have used two closely related non-model species of Geranium to study the contrasting effects in shade tolerant vs shade avoiding plants. They have analysed the involvement of elongation stimulating hormones and came to the conclusion that in the shade avoiding plants auxin and GA biosynthesis are responsible for the effect. Furthermore, evidence for local light perception and subsequent elongation in the petioles is presented. The subject is introduced adequately, and the manuscript is well written. There are however some points that need clarification and/or experimentation to make the findings more sound. I have detailed this below.

Methods:

-line 140: what sort of light sources are used to produce "white" light

We have added the brand of the lamps used in our growth chambers, which produce the white light background, to the methods section. These are Philips HPI 400 W and similar to those used in Gommers et al., 2017 (The Plant Cell).

-line 145: the light bundles: how are they applied on petiole and lamina? Are they covering the entire petiole or lamina, or a spot with a certain area?

We have specified the use of the light bundles and the specific treated part of the leaves in the method section. For petioles the apical half was illuminated, for lamina's the entire lamina was illuminated. Setups used were modified from Pantazopoulou et al., 2017 (PNAS).

-in hormone analysis and pharmacological treatments the provenance of the majority of the compounds is not communicated. Please include this information.

We have added the information about suppliers and/or references for the used chemicals.

Results

-re-analysis of the transcriptome dataset can be more catalogued. Mention which GO terms were overrepresented in a list, and at which position auxin, GA and BR come. Also mention in the list which is the expectancy in case of random regulation.

We understand the question of the reviewer about a more extended GO enrichment analysis. Nevertheless, this RNAseq study was published before, and complete lists of the over-represented GO categories for both species at both time-points are available in Gommers et al. 2017 (The Plant Cell). To not reproduce the same data, and to focus on hormone patterns, we present here all auxin, GA and BR associated GO clusters, and the level of over representation among up- and down- regulated genes upon WL+FR in the two species. We chose to present the LOG of the p-value of the enrichment tests (GO-seq), because this indicates if a GO term is over-represented among differentially expressed genes compared to what would be expected compared to random distribution. This is the key piece of information, and we have highlighted the GO terms with a p-value < 0.05 (-Log(p-value) > 2) by adding an asterisk. This is a commonly used representation of GO-seq data (optimized GO analysis for RNAseq data). We have, nevertheless, made it more prominent in the caption that these GO enrichment scores are derived from Gommers et al., 2017, where also the necessary methodological details are available (line 242-243).

-Figure 2 contains data directly derived from the transcriptome. The authors should validate these data by qPCR of regulated genes, at least those that may directly relate to changes in measured hormone level.

We have added the expression of auxin synthesis, auxin signaling, GA synthesis and BR signaling genes, as measured in a separate experiment by RT qPCR (Fig. 3). We refer to this data in the paragraph starting at line 248.

-line 256: the authors focus on GA1, while other active GAs may also be of importance. They should discuss on the levels of the active GA5, which go up in FR in petioles in the shade tolerant plant. Also, it is not clear what other GAs (including GA4 and GA7) were doing. Were they below detection limit? this could be mentioned.

First, indeed, the GA's not presented in the manuscript were below the detection limit in these species. We have mentioned this for GA_4 and GA_7 in line 285-288.
Also, we have made a statement for GA_5, which appears to be undetectable in *G. robertianum* petioles in WL but abundant in WL +FR. We have no direct explanation for the increase of GA_5 levels but can conclude that it apparently does not induce shade avoidance (now mentioned in the manuscript) in *G. robertianum*. To support this last statement, we have added a reference to an article in which also a biological active GA (GA_1) was increased in a non-shade avoiding mutant of *Arabidopsis* (Li *et al.*, 2016) (line 356-361).

-the hormone treatments work (line 282 and further) in robertianum, while the inhibitor treatments do not in pyrenaicum (while presumably the trichome density is very similar). The authors switch to seedling use because of this. An experiment, where this switch would not be necessary, consisting of studying excised leaves or petioles will help. If local light perception also directs local hormone content, this system should work for the FR elongation

assay and the associated pharmacological treatments, and may be a better proxy of what happens in true leaves, than studying effects at seedling stage.

Indeed, the contrasting effects of synthetic hormones (strong effect) and hormone inhibitors (no effect) on *G. pyrenaicum* petioles is hard to explain. We do know that these inhibitors are effective in this species, based on the seedling data. Therefore, we hypothesized that the problem lies with the application of the chemicals to leaf tissue. Possibly, the chemicals do not enter the petiole well across the epidermis. The synthetic hormones can, in that case still affect epidermal cell (and thus petiole) elongation, but the inhibitors might not be able to affect hormone synthesis or perception that could also occur in other cell layers.

Although we had also considered excised leaves, we preferred a system that leaves the organism intact, thus preventing damage-associated side-effects and partially submerging the petioles in solution, which also causes side-effects. We believe that seedlings in vertical agar plates represent a fair system to test the involvement of these hormones in FR-induced growth in *G. pyrenaicum*. Although it is not identical to 2-week old rosette plants, the site of shade-induced growth is comparable (the cotyledon petiole vs. the leaf petiole).

-line 333: changes in hormone abundance start later than gene expression. Yes, but please provide some explanation why. This would be normal for biosynthesis genes, but what about genes that are considered reporters for the signaling pathway?

We have added a sentence which states our hypothesis concerning the timing of growth and detectable hormone levels. We state that the early induced growth could be due to increased sensitivity to the already present levels of hormones, which is regulated by the expression of genes encoding proteins in the different hormone-signaling pathways (similar to Arabidopsis). After prolonged shade exposure, growth would be maintained by increased levels of hormones. (lines 363 – 371)

-line 372: please compare/contrast your findings with findings in other species (in literature, beyond Arabidopsis) and discuss.

As requested, we have included two new references, to a study with FR-exposed sunflower, and dark-grown pea, in which CS levels were also lower or unchanged compared to the light control. (lines 417 – 420)
